# Structural Basis of Membrane Trafficking by Rab Family Small G Protein

**DOI:** 10.3390/ijms14058912

**Published:** 2013-04-25

**Authors:** Hyun Ho Park

**Affiliations:** School of Biotechnology and Graduate School of Biochemistry, Yeungnam University, Gyeongsan 712-749, Korea; E-Mail: hyunho@ynu.ac.kr; Tel.: +82-53-810-3045; Fax: +82-53-810-4769

**Keywords:** membrane trafficking, ras-superfamily, small G protein, rab GTPase, protein structure

## Abstract

The Ras-superfamily of small G proteins is a family of GTP hydrolases that is regulated by GTP/GDP binding states. One member of the Ras-superfamily, Rab, is involved in the regulation of vesicle trafficking, which is critical to endocytosis, biosynthesis, secretion, cell differentiation and cell growth. The active form of the Rab proteins, which contains GTP, can recruit specific binding partners, such as sorting adaptors, tethering factors, kinases, phosphatases and motor proteins, thereby influencing vesicle formation, transport, and tethering. Many Rab proteins share the same interacting partners and perform unique roles in specific locations. Because functional loss of the Rab pathways has been implicated in a variety of diseases, the Rab GTPase family has been extensively investigated. In this review, we summarize Rab GTPase- mediated membrane trafficking while focusing on the structures of Rab protein and Rab-effector complexes. This review provides detailed information that helps explain how the Rab GTPase family is involved in membrane trafficking.

## 1. Introduction

**Figure 1:**
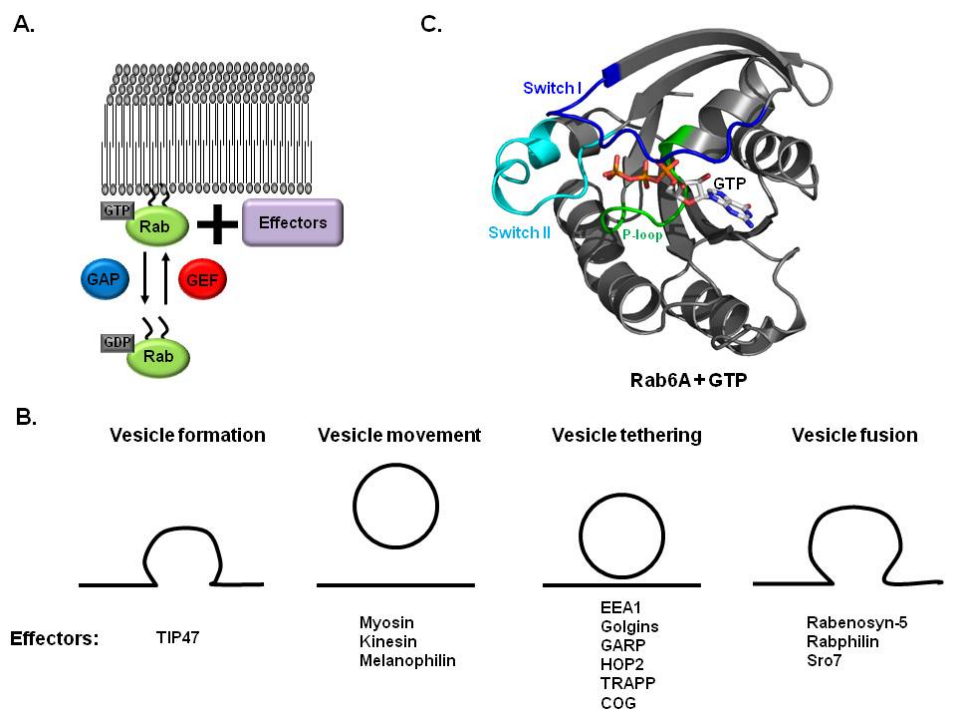
(**A**) Schematic model of the nucleotide and membrane recruitment cycles of Rab GTPases; (**B**) Involvement of Rab effectors during different stages of membrane trafficking. Rabs can properly execute their various functions by recruiting specific effectors during membrane trafficking; (**C**) Crystal structure of the Rab6A/GTP complex (PDB ID: 2GIL). GTP is shown as a stick model with colors. P-loop: phosphate binding loop formed by GXXXXGKS(T); Switch I: corresponding amino acid 38–49; Switch II: corresponding amino acid 69–81). Switch I and switch II are importance for GDP-GTP exchanged mediated activity control.

Small GTPase proteins including the Ras-superfamily of small G proteins are involved in membrane trafficking [1–4]. The Ras-superfamily is composed of three subfamilies, the Rab family, Arf/Arl/Sar family, and Rho family. GTP hydrolysis activity of the Ras-superfamily is regulated by GTP/GDP binding states [3,5]. One Ras-superfamily, Rab, is known to be involved in the regulation of vesicle trafficking, which is critical to endocytosis, biosynthesis, secretion, cell differentiation and cell growth [2,6–8]. The active form of Rab proteins, which contains GTP, can recruit specific binding partners such as sorting adaptors, tethering factors, kinases, phosphatases and motor proteins, and influence vesicle formation, transport, and tethering [9–12]. Many Rab proteins share the same interacting partners and play unique roles in specific locations [13]. Rab functions as a molecular switch by changing between a GDP-bound inactive state and a GTP-bound active state [14]. Changing between GDP and GTP is controlled by guanine nucleotide exchange factors (GEFs) and GTPase activating proteins (GAPs) [4,15–17]. GAP activates hydrolysis of GTP in Rab and makes Rab GDP binding inactive. GDP in inactive Rab can be replaced with GTP via GEF. The GTP-bound active form of Rab can be recruited to membrane vesicles, where it promotes membrane trafficking by interacting with specific effector proteins (Figure 1A) [18,19]. Although a few effectors can interact with a GDP-bound inactive form of Rab, most effectors bind to GTP-bound active form of Rab and are involved in membrane trafficking [20]. Many Rab effectors are identified so far. They function at different stages of membrane trafficking: Budding, transporting, tethering, and fusion (Figure 1C). The function of TIP47 with Rab9 is the best studied case of Rab and effector working at vesicle formation stage. The interaction between TIP47 and Rab9 leads to recruitment of mannose-6-phosphate receptor to TIP47 causing the formation of the transport vesicle [21]. At the vesicle movement stage, the well known case is the interaction of Rab11 with its effector, Rab11 family interacting protein 2 (Rab11-FIP2). Rab11-FIP2 bound Rab11 can interact to myosin Vb, which causes the movement of vesicle [22]. Many membrane tethering factors such as golgins, which are coiled-coil tether proteins, and TRAPP I, II, and COG complex, which are multisubunit tethers, are also interact to various Rab family during vesicle tethering stage of membrane trafficking [23,24]. In addition to working with many tethering effectors at vesicle tethering stage, Rab proteins also are involved in regulation of SNARE protein dependent fusion of vesicles. Rabs can either interact directly with SNARE protein or with proteins that regulate SNARE activity during fusion stage of membrane trafficking.

More than 60 Rab isoforms have been identified in humans, but the function of each isoform is poorly understood. Rab1, Rab5, Rab6, Rab7, and Rab11 are known as housekeeping Rabs, which are conserved from yeast to humans [2,10]. Because a functional loss of the Rab pathway has been implicated in a variety of human diseases, the Rab GTPase family has been extensively studied [9,25–30]. In this review, we summarize Rab GTPase-mediated membrane trafficking while focusing on the structures of the Rab protein and Rab-effector complexes to provide a detailed understanding of how the Rab GTPase family is involved in membrane trafficking.

## 2. Rab and Membrane Trafficking

### 2.1. Rab GTPases Involved in Membrane Trafficking

Over 60 members of the Rab family have been identified in humans. Initially, proteomic studies and studies of the expression of green fluorescent protein (GFP)-tagged protein showed that around 14 members of the Rab family, including Rab3A/B/C/D, Rab4A, Rab8B, Rab11A/B/C, Rab26, Rab27A/B, Rab33A, and Rab37, are involved in membrane trafficking [7,10,13]. The majority of trafficking-related Rabs function in housekeeping mode, except for Rab4A (endosome-resident), Rab8B (Golgi-resident), and Rab11B (endosome-resident), which work in specific places.

### 2.2. Rab, GEF and GAP in Membrane Trafficking

The activity of Rab GTPase is regulated by the transition between the GDP-bound state (inactive form) and GTP-bound state (active form). To ensure the proper role of the Rab family during membrane trafficking, GDP exchange with GTP and GTP hydrolysis have to be controlled rapidly. Although the GDP releasing process in the Rab family occurs spontaneously at an extremely slow rate, this process is usually accelerated by guanine nucleotide exchanging factor (GEF) [16,31]. The GEF interaction with the Rab family leads to structural changes in Rab, especially nucleotide binding sites in the switch I and switch II regions. Specifically, the switch I and switch II regions are opened and Mg2+ is displaced upon binding of GEF, which causes weakening of the binding affinity of GDP in the Rab family (Figure 1B) [16,32–34].

GTP hydrolysis activity of the Rab family is extremely slow in cells and is activated by an additional Rab interacting protein, GTPase activating protein (GAP) [35–38]. The activation mechanism upon binding of GAP to Ras family of small G protein is firstly studied by Ras:RasGAP complex structure [39–41]. Several GEFs and GAPs specifically work on Rab proteins have been identified and summarized in Table 1.

## 3. Structures of Rab and Its Effector Complex

### 3.1. Structure of Rab Subfamily

The first structure of the small GTPase protein was revealed in the H-Ras protein in 1989 [42]. This structure consists of a central 6 stranded mixed β-sheet surrounded by 5 α-helices. The Rab family also exhibited the typical structure first seen in H-Ras (Figure 1B) [43–45]. Five conserved sequence motifs involved in nucleotide binding were identified in the structure of Ras and the Rab family. The P-loop, which is a well known nucleotide binding motif that contains consensus sequence GXXXXGKS(T), was conserved and located at amino acids 20–27 in Rab6A (Figure 1B). Switch I (amino acids 38–49 in Rab6A) and switch II (amino acids 69–81 in Rab6A) were also identified in the Rab family (Figure 1B). Structures of these switch regions changed most significantly upon binding of nucleotides and were shown to be important to regulation of the activity of the Rab family. The N/TKCD motif functions to discriminate against other nucleotides and was also identified in the Rab family.

### 3.2. Structure of Rab/GAP Complex

**Figure 2:**
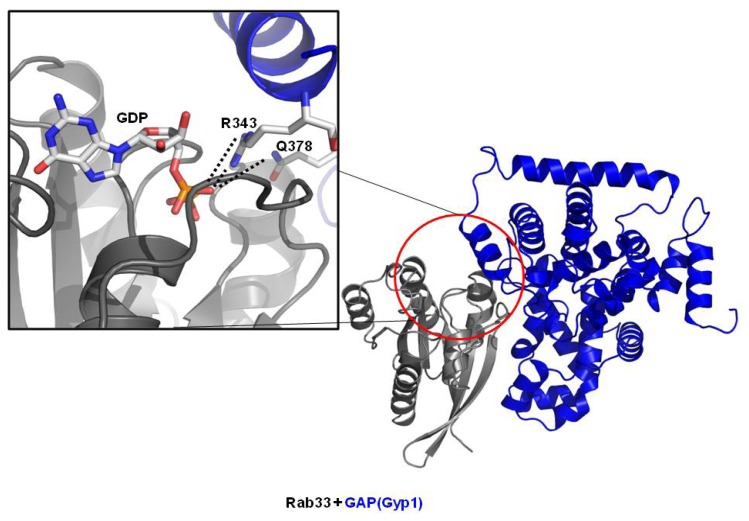
Crystal structure of Rab33/Gyp1 complex (PDB ID: 2G77)—The representative Rab/GAP complex structure. Close-up view shows the involvement of arginine finger and glutamine finger in the interaction with GDP. R343 is arginine finger and Q378 is additional glutamine finger. Salt bridges are shown by dotted line.

GAP proteins contain a conserved TBC (Tre2/Bub2/Cdc16) domain that is responsible for the GAP activity in the Rab family. All characterized Rab GAP proteins identified to date contain the TBC domain. The working mechanism of GAP was initially described based on the crystal structure of the TBC domain of Gyp1, which is the GAP for Ypt1 [46]. The arginine finger on the TBC domain of Gyp1 was faced with the Rab nucleotide binding pocket, which stimulates GTPase activity of the Rab family. The additional glutamine finger responsible for stimulating the activity of the Rab family was found in the crystal structure of the Rab33/Gyp1 complex (Figure 2) [41]. Both fingers from GAP protein contribute to stabilize the β-phosphate of GTP so that γ-phsphate of GTP easily hydrolyze and dissociate from Rab. The working mechanism of GAP on the Rab family appears to be conserved and is expected to be the same as that of GAP proteins for Ras, despite structural differences.

### 3.3. Structure of Rab/GEF Complex

**Figure 3:**
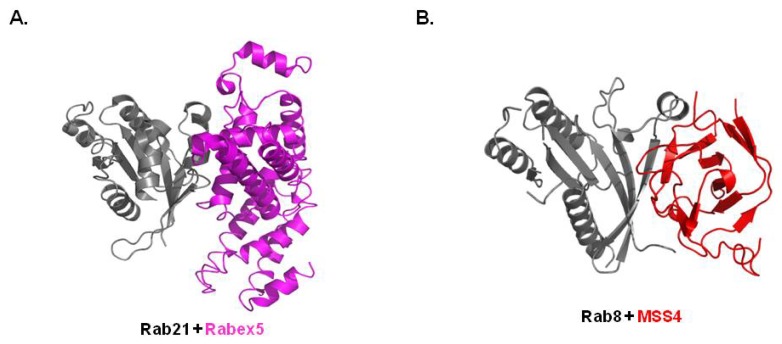
(**A**) Crystal structure of Rab21/Rabex5 complex (PDB ID: 2OT3)—The representative Rab/GEP complex structure; (**B**) Crystal structure of Rab8/MSS4 complex (PDB ID: 2FU5)—The representative Rab/GEP complex structure.

Due to sequence diversities and a lack of clear motifs that define RabGEF, few RabGEFs have been identified to date. Rabex-5 is a well-known RabGEF that functions in members of the Rab5 subfamily including Rab5a,b,c, Rab21, Rab22a and Rab22b. Vps9 domain in Rabex-5 is responsible for the interaction with the Rab5 subfamily. The structure of the Rab21 and Vps9 domain of the Rabex-5 complex revealed that aspartic acid from the Rabex-5 Vps9 domain interacts tightly with positively charged lysine from the Rab21 P-loop, disrupting nucleotide binding and stabilizing the nucleotide-free state (Figure 3A) [34]. The P-loop lysine is conserved and critical for high affinity nucleotide binding in the Rab family. The recent crystal structure of the Rab8/MSS4 (Mammalian Suppressor of SEC4) complex revealed diversity in the mechanism of Rab nucleotide exchange when compared with the working mechanism of Rabex-5 on Rab21. The structure of the Rab8a/MSS4 complex showed that MSS4 binds to Rab8 via its switch I and interswitch domain (Figure 3B) [33]. MSS4 has also been shown to interact with many other Rab proteins via both exocytic and endocytic pathways. Due to the binding capacity and lack of specificity of MSS4, the general chaperone function of MSS4 rather than a specific GEF was suggested. DENN-domain was identified as another Rab binding domain of RabGEF recently [47]. A recently solved Rab35/DENN domain of DENND 1B-S complex structure indicated that bi-lobed DENND1B-S directly interact to Rab35. This interaction leads conformational changes in switch I of Rab, which causes the nucleotide-binding pocket open and makes easy to exchange from GDP to GTP [48].

### 3.4. Structure of Rab/Other Effector Complex

Rab small G proteins perform their activity with many effector molecules that are often unrelated in sequence and structure [12,49–52]. Rab small G protein in its active form (GTP-binding state) can recruit effector proteins. In general, GTP binding to Rab protein induces rigid switch regions, revealing a hydrophobic patch that interacts with the Rab-binding domain (RBD) of effector proteins [42,52,53]. Effector proteins are usually multi-domain proteins containing additional domains that mediate biological functions [17,54]. Because structural studies of Rab6 and its effectors have led to its being well-characterized among Rab proteins, we will focus on and discuss recent structural studies of Rab6 and its effector complexes.

**Figure 4:**
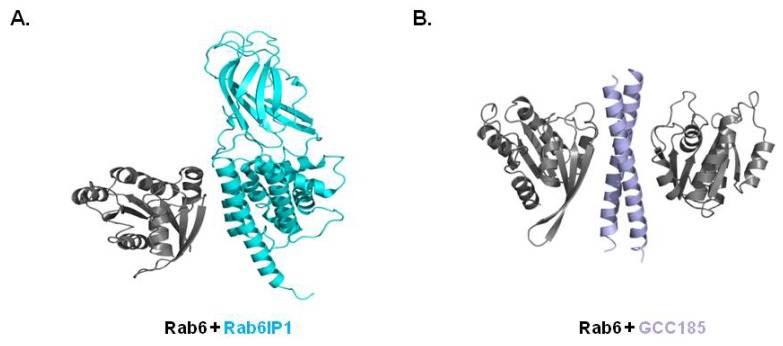
(**A**) Crystal structure of the Rab6/Rab6IP1 complex (PDB ID: 3CWZ)—The representative Rab6/effector complex structure; (**B**) Crystal structure of Rab6/GCC185 complex (PDB ID: 3BBP)—The additional 2:2 complex structure of the Rab6/effector.

Rab6 regulates membrane trafficking at the level of the Golgi and can be divided into three isoforms, Rab6A, Rab6B, and Rab6C (Rab6A′) [11,43,55]. Genetic and biochemical studies have revealed many effector proteins that work with Rab6 via tight interactions including dynein, Rabkinesin, Rab6IP1 (Rab6 interacting protein1), Rab6IP2 (Rab6 interacting protein2), TMF (TATA element modulatory factor), mint3, Bicaudal D2, p150, and PIST (protein interacting specifically with TC10) [2,10,51–57]. Currently, only a few complex structures between Rab6 and its effectors are available including Rab6:Rab6IP1 [54] and Rab6:GCC18 [58]. All available complex structures have revealed truncated effector domains encompassing RBD. Indeed, no structural information describing the full-length effector protein in complex with Rab protein have been reported. Two complex structures revealed that one or two α-helices from the RBD of the effector proteins directly interact with switch I and switch II of Rab6 (Figure 4A,B).

The crystal structure of the Rab6a:Rab6IP1 complex showed that switch I, switch II and the inter-switch region of Rab6 interact with the first and eighth α-helices of the RUN domain of Rab6IP1. The fragments of Rab6IP1 containing the RUN domain and PLAT domain were co-crystallized with Rab6A and solved the structure. The greatest contributing interaction between the two proteins was that of the hydrophobic interactions [54]. Hydrophobic triads (Phe50, Trp67, Tyr82), Ile46, Phe75, and Leu78, from switch I, switch II and the inter-switch of Rab6A were involved in the hydrophobic interaction. Strong salt bridges formed by Lys13 of Rab6 and Asp901 of Rab6IP1 were also detected. Unlike the 1:1 complex of Rab6:Rab6IP1, Rab6:Gcc185 complex was formed as a 2:2 complex (Figure 4B). The α-helix of GCC185 dimerizes and interacts with two Rab6 in a 2:2 complex. This dimerization based interaction mechanism has also been detected in Rab11 [59]. Two Rab11 molecules interact with the dimerized α-helix of FIP2, which is a well-known effector of Rab11 [59]. The characteristics of the interface of both complex structures are almost identical, although the overall structures are different (Figure 4). The central hydrophobic core is the main force for the interaction. Extra salt bridges were only detected in the Rab6:Rab6IP1 complex. Although the overall interactions are similar among both complex structures, the hydrophobic triad formed by Phe50, Trp67, and Tyr82 from Rab6 exerts two distinct conformations in the complex structures. This conformational diversity of switch I and switch II of Rab6 is necessary for adaptation of two unrelated hydrophobic surfaces formed by Rab6IP1 and Gcc185. The conformational diversity of the hydrophobic triad was also detected in different subfamilies of Rabs.

## 4. Conclusions

Information describing Rab function in membrane trafficking has increased. More than 60 types of Rab proteins perform unique roles in the endocytic and exocytic membrane trafficking pathways at distinct locations in the cell. Recently identified Rab proteins and studies of their localization have helped elucidate how Rab family members communicate with each other during membrane trafficking. The structure of Rab and many interacting proteins including GAP, GEF, and effector proteins and their complexes have provided critical information showing how they participate in membrane trafficking. Although the structures of many Rab proteins are similar, the interaction partners and their downstream functions are totally different.

Rab proteins are known to be linked to many pathologies of a wide range of human diseases from cancers to neurological diseases (Table 2). Several Rab proteins including Rab25 have been implicated in multiple cancers. Rab proteins are also involved in neurological diseases such as Parkinson’s and Huntington’s diseases. Because of the unique polarized structure and function of neurons, they are more sensitive to abnormalities of membrane trafficking. It is well known that Rab3 is important to synaptic function, that Rab11 and Rab13 are important for neurite growth and membrane remodeling, and that Rab23 is critical to general nervous system development. Because of the critical role of Rab proteins in membrane trafficking and their association with many human diseases, further structural and biochemical studies of Rab and Rab/effector complexes are critical to fully understand the biological roles and involvement of diseases of the Rab family and Rab recruitment during membrane trafficking.

## Figures and Tables

**Table 1:** A few examples of Rab-guanine nucleotide exchange factors (GEFs) and Rab-GTPase activating proteins (GAPs) and their target Rabs.

GEF		GAP	
	
Name of GEF	Target Rab	Name of GAP	Target Rab
RABIF	Rab3A, Rab8	TBC1D2	Rab7
RAB3IP	Rab3A	TBC1D3C	Rab5
RABGEF1	Rab5, Rab22	TBC1D13	Rab35
RIN1	Rab5	RUTBC1	Rab 9A
ANKRD27	Rab32, Rab38	RAB3GAP	Rab3

**Table 2:** A few examples of disease-related Rab family and associated diseases.

Rab family	Membrane traffic pathway	Associated diseases	References
Rab3	Exocytosis, neurotransmitter release	Warburg micro syndrome (Rab3GAP mutation), Martsolf syndrome (Rab3GAP mutation)	[60,61]

Rab6	Golgi mediated vesicle transport	Gerodermia osteodysplastica (Rab6 effector Golgin mutation)	[62]

Rab7	Late endosome to lysosome	Charcot-Marie-Tooth	[63]

Rab8	Exocytosis, TGN to plasma membrane	Bardet-Biedel syndrome, Huntington’s disease	[64]

Rab11	TGN to plasma membrane	Huntington’s disease	[30,65]

Rab23	Protein transport to plasma membrane	Carpenter syndrome	[66]

Rab25	RE to Plasma membrane	Epithelial cancers	[67]

Rab27	Exocytosis	Griscelli syndrome	[68]
